# Nurse‐Led Foot‐Ankle Exercises and Changes in Neuropathy Symptoms and Diabetic Foot Risk Indicators: A Quasiexperimental Study

**DOI:** 10.1155/nrp/9196936

**Published:** 2026-04-20

**Authors:** Yasmin Ibrahim Abdelkader Khider, Rasha Fathy Ahmed Dawood, Heba Mohammed Mahmoud Elhapashy, Heba Mahmoud Mahmoud Mohamed, Fatma M. Ibrahim, Shaimaa Mohamed Elghareeb Allam

**Affiliations:** ^1^ Medical-Surgical Nursing Department, Faculty of Nursing, Mansoura University, Mansoura, Egypt, mans.edu.eg; ^2^ Faculty of Nursing, Mansoura National University, Mansoura, Egypt; ^3^ Medical-Surgical Nursing Department, Faculty of Nursing, Alexandria University, Alexandria, Egypt, alexu.edu.eg; ^4^ Gerontological Department, Faculty of Nursing, Mansoura University, Mansoura, Egypt, mans.edu.eg; ^5^ RAK College of Nursing, RAK Medical and Health Sciences University, Ras Al Khaimah, United Arab Emirates

**Keywords:** diabetes mellitus, diabetic foot, diabetic peripheral neuropathy, foot-ankle exercise, risk assessment

## Abstract

**Aim:**

To examine whether a nurse‐led foot‐ankle flexibility and resistance exercise programme, delivered alongside usual care, was associated with changes in neuropathy symptoms and diabetic‐foot risk/self‐care indicators among adults with diabetes.

**Background:**

Diabetic peripheral neuropathy (DPN) contributes to reduced protective sensation, limited joint mobility and impaired self‐care, increasing DFU risk. Low‐cost exercise programmes targeting foot‐ankle mobility and strength may improve neuropathy‐related symptoms and modifiable DFU risk factors, but practice‐based evidence in routine nursing settings remains limited.

**Design:**

Quasiexperimental, nonrandomized, nonequivalent control‐group pre–post study.

**Methods:**

Adults with Type 1 or Type 2 diabetes attending an outpatient diabetes clinic were recruited by convenience sampling (*N* = 60) and allocated to an intervention group (usual care plus structured exercise education and supervised practice; *n* = 30) or a control group (usual care; *n* = 30). Outcomes were assessed at baseline and 1 month using the Neuropathy Total Symptom Score‐6 (NTSS‐6) and the Diabetes Foot Assessment/Risk Screening scale. Because allocation was nonrandomized and outcomes were analysed in categories, inferential analyses were exploratory and based on chi‐square/Fisher’s exact tests with Cramer’s V effect sizes.

**Results:**

At 1 month, neuropathy symptom severity categories differed significantly between groups (*χ* ^2^ = 34.236 and *p* < 0.001; V = 0.76), with a greater proportion of participants in the intervention group shifting to lower symptom‐severity categories. Diabetes Foot Assessment/Risk Screening categories also favoured the intervention group (*χ* ^2^ = 30.762 and *p* < 0.001; V = 0.72). Given the quasiexperimental design and unadjusted categorical analyses, these findings should be interpreted as associations rather than causal estimates.

**Conclusions:**

In this practice‐based quasiexperimental study, a brief nurse‐led foot‐ankle flexibility and resistance exercise programme was associated with improved neuropathy symptom categories and more favourable diabetic‐foot risk/self‐care indicators over 1 month. The findings support feasibility within nursing practice, but causal inference is limited by nonrandom allocation, the use of categorical unadjusted analyses and the use of a study‐specific foot‐risk/self‐care measure.

**Relevance to Clinical Practice:**

Nurses can integrate brief foot screening with structured foot‐ankle exercise education and demonstration during outpatient diabetes visits as an adjunct to, not a replacement for, standard diabetic‐foot surveillance and self‐management support.

**Summary**
• No Patient or Public Contribution
◦ Patients, service users, caregivers or members of the public were not involved in the design, conduct, analysis or interpretation of the study, or in preparation of the manuscript. This work was designed and delivered within routine outpatient clinic care, and participant involvement was limited to enrolment and provision of study data.
• Implications for Practice
◦ A short, nurse‐led foot‐ankle exercise package can be delivered in outpatient diabetes clinics using brief education, supervised practice and simple printed materials as an adjunct to routine foot screening and standard preventive education.
◦ Routine follow‐up (e.g., telephone calls) may support adherence and reinforce daily foot self‐care behaviours.
◦ Foot‐risk screening should be combined with tailored education, teach‐back and reinforcement, particularly for patients who may need additional support with health literacy.

## 1. Introduction

Diabetes is a growing global health challenge, with most adults living with the condition residing in low‐ and middle‐income countries [1]. Diabetic peripheral neuropathy (DPN) is a common complication and a major contributor to diabetic foot ulceration through loss of protective sensation, altered foot biomechanics and reduced ability to detect early tissue injury [2, 3]. Diabetic foot ulcers are associated with infection, hospitalization, amputation, reduced quality of life and high costs [4, 5].

Recurrence after healing is common, underscoring the need for sustained risk reduction and self‐management [6]. Across settings, diabetic foot complications remain prevalent [7]. In addition to medical management, prevention strategies rely on early identification and management of modifiable risk factors, including limited ankle and foot joint mobility, intrinsic muscle weakness, abnormal plantar loading and deficits in foot self‐care [8, 9]. Current guidance recommends routine screening, education, appropriate footwear and targeted exercise interventions to improve mobility and function in people at low‐to‐moderate ulcer risk [3, 8].

Exercise is a cornerstone of diabetes management and is recommended in clinical guidance for adults with diabetes [10]. Foot‐ankle exercise programs that combine flexibility and resistance training have shown promise for improving neuropathy‐related symptoms, intrinsic foot muscle strength, and biomechanical risk factors [11–13]. However, systematic reviews indicate that evidence is mixed regarding effects on first ulcer occurrence, highlighting the need to focus on measurable intermediate outcomes and feasibility in real‐world clinical settings [14–16].

Technologies such as wearable sensors may support early ulcer detection [17], yet scalable prevention in many settings also depends on low‐cost, nurse‐deliverable strategies that strengthen self‐care and address modifiable risk factors. Nurses are central to diabetes education, risk screening and self‐management support; therefore, a structured nurse‐led foot‐ankle exercise programme may be a practical intervention within routine outpatient care.

Therefore, this study aimed to examine whether foot‐ankle flexibility and resistance exercises, delivered by nurses in routine outpatient care, were associated with changes in neuropathy symptoms and diabetic‐foot risk indicators among adults with diabetes. Given the nonrandomized design, outcomes were conceptualised as intermediate clinical indicators rather than direct evidence of ulcer prevention.

## 2. Methods

### 2.1. Design

This study used a quasiexperimental, nonequivalent control‐group design with pre‐ and postintervention assessments. Reporting was guided by recommendations from the Transparent Reporting of Evaluations with Nonrandomized Designs (TREND) statement [18]. Because the intervention was delivered pragmatically within routine care, the study should be interpreted as a practice‐based evaluation rather than a causal efficacy trial.

### 2.2. Setting

The study was conducted in the diabetes outpatient department of a university hospital in Egypt. Data collection occurred from April 2024 to December 2024.

### 2.3. Participants and Recruitment

Participants were recruited using convenience sampling from adults attending the clinic for diabetes follow‐up. Eligibility criteria included (a) diagnosis of Type 1 or Type 2 diabetes, (b) age 20–60 years, (c) receiving regular diabetes treatment, (d) no current signs or symptoms of a diabetic foot ulcer at enrolment and (e) ability to be contacted by telephone for follow‐up. Exclusion criteria included history of severe mental illness, acute neurological disorders (e.g., epilepsy), cardiopulmonary dysfunction or other conditions that would limit safe participation in exercise and prior structured instruction in foot‐ankle exercise.

### 2.4. Sample Size

A priori power analysis was performed using G ∗ Power (v3.1.9.7) [19]. Assuming a large between‐group effect (*d* = 0.80), two‐tailed alpha = 0.05 and 80% power, the minimum required sample was 26 participants per group (*N* = 52). The final analysed sample comprised 60 participants, with 30 participants in each group, which exceeded the minimum required sample.

### 2.5. Allocation

Because randomization was not feasible in this clinical setting, eligible participants were allocated to intervention or control groups based on practical considerations (e.g., clinic scheduling) to minimize contact between groups and reduce contamination. This pragmatic allocation strategy may have introduced selection bias and residual confounding; therefore, baseline differences were examined and results are interpreted cautiously.

### 2.6. Intervention

The intervention consisted of usual care plus a nurse‐led foot‐ankle flexibility and resistance exercise programme delivered over five sessions: one theoretical session and four practical sessions. Sessions were delivered individually or in small groups (30–45 min each) using simplified Arabic language. Educational content addressed diabetic foot ulcer risk factors and prevention, and practical content focused on demonstrating and rehearsing active range‐of‐motion and strengthening exercises targeting the ankle and foot. Participants were provided with an illustrated booklet and were encouraged to practise the exercises at home during the 1‐month follow‐up period. Telephone follow‐up was used to support adherence and answer questions.

### 2.7. Control Condition

Participants in the control group received usual care provided by the clinic, including routine medical follow‐up and standard diabetes education and foot care advice.

### 2.8. Measures

Data were collected using three instruments. (1) A researcher‐developed biosociodemographic and clinical questionnaire (e.g., age, sex, education, duration of diabetes and hospitalization history), informed by prior work [11, 20]. (2) The Neuropathy Total Symptom Score‐6 (NTSS‐6), a validated self‐report measure assessing six DPN sensory symptoms [21]. Total scores were categorised to reflect symptom severity [22]; although this aided clinical interpretation, categorisation may have reduced measurement granularity. (3) A Diabetes Foot Assessment/Risk Screening scale developed from established diabetic‐foot risk constructs [2, 23]. The tool includes 10 yes/no items assessing foot risk factors and self‐care; it demonstrated acceptable internal consistency (Cronbach’s alpha = 0.82) and content validity based on expert review. Because this scale was developed for the present study, its use should be regarded as pragmatic and exploratory; construct validity, test–retest reliability and responsiveness were not established.

### 2.9. Procedures

After informed consent, baseline assessments were completed face‐to‐face for both groups. The intervention group then received the exercise programme in addition to usual care. Postintervention assessments were conducted at 1 month for both groups using the same outcome measures.

### 2.10. Data Analysis

Data were analysed using IBM SPSS Statistics (Version 20.0). Descriptive statistics summarised participant characteristics. Between‐group differences at baseline and at 1 month were examined using Pearson chi‐square or Fisher’s exact tests as appropriate. Effect sizes for categorical outcomes were quantified using Cramer’s V. Because group allocation was nonrandomized and outcomes were analysed as categories, the inferential analyses should be considered exploratory and unadjusted; findings were, therefore, interpreted with emphasis on direction and magnitude of association rather than causal attribution. Statistical significance was set at *p* < 0.05 (two‐tailed).

### 2.11. Ethical Considerations

Ethics approval was obtained from the Institutional Review Board of the Faculty of Nursing at a university in Egypt (approval number masked for peer review). All participants provided written informed consent. Confidentiality and the right to withdraw without consequences were assured, and procedures were conducted in accordance with the Declaration of Helsinki (see Table 1).

**TABLE 1:** Intervention description (TIDieR summary).

Name	Nurse‐led foot‐ankle flexibility and resistance exercise programme
Why	To improve foot‐ankle mobility, strength and self‐care behaviours relevant to neuropathy symptoms and DFU risk.
Materials	Illustrated Arabic booklet/handout; chair for seated practice; optional elastic resistance band for strengthening; telephone follow‐up script.
Procedures	One theory session (DFU risk and prevention) + four practical sessions demonstrating and rehearsing exercises; home practice encouraged.
Who provided	Registered nurse(s) from the diabetes clinic, oriented to the intervention protocol and supported by a delivery checklist to promote consistency.
How	Delivered individually or in small groups (30–45 min), using simplified Arabic language and teach‐back.
Where	Outpatient diabetes clinic education/practice area.
When and how much	Five sessions (1 theory + 4 practical; 30–45 min each) during enrolment, plus home practice for 1 month. Home practice was recommended at least 5 days/week for 15–20 min/session, progressed according to comfort and tolerance.
Tailoring/adherence	Instructions tailored for health literacy using teach‐back; session attendance recorded; scheduled telephone calls used to reinforce home practice and address barriers.

### 2.12. Outcome Measures

Neuropathy symptoms were assessed using the NTSS‐6. Diabetic‐foot risk/self‐care was assessed using a 10‐item yes/no screening scale. Each item was scored 0‐1 and summed (range 0–10), with higher scores indicating greater DFU risk and poorer preventive self‐care. Total scores were categorised into lower risk, higher risk and self‐care knowledge deficit according to the study scoring guide to support clinical interpretation. Because the scale was study‐specific and summarised categorically, this outcome should be interpreted as a practical risk/self‐care indicator rather than a definitive clinical risk classification.

## 3. Results

### 3.1. Participant Characteristics

The analysed sample comprised 60 participants, with 30 in the control group and 30 in the intervention group. Baseline sociodemographic and clinical characteristics were comparable between groups (Table 2). No statistically significant between‐group differences were observed for age (*χ* ^2^ = 0.287 and *p* = 0.592), sex (*χ* ^2^ = 0.089 and *p* = 0.766), educational level (*χ* ^2^ = 0.530 and *p* = 0.767), occupation (*χ* ^2^ = 0.530 and *p* = 0.767), previous hospitalization (*χ* ^2^ = 0.162 and *p* = 0.688), duration of diabetes (*χ* ^2^ = 1.017 and *p* = 0.313), regular medication use (*χ* ^2^ = 0.218 and *p* = 0.640) or family history (*χ* ^2^ = 0.218 and *p* = 0.640). Most participants in both groups were aged 50–60 years, male, married and had a secondary education level.

**TABLE 2:** Baseline sociodemographic and clinical characteristics by group.

Variable	Category	Control *n* (%)	Intervention *n* (%)	*χ* ^2^	*p*
Age	40–50 years	12 (40.0)	10 (33.3)	0.287	0.592
	50–60 years	18 (60.0)	20 (66.7)		

Sex	Male	23 (76.7)	22 (73.3)	0.089	0.766
	Female	7 (23.3)	8 (26.7)		

Marital status	Married	30 (100.0)	30 (100.0)	—	—

Educational level	Higher education	11 (36.7)	13 (43.3)	0.530	0.767
	Secondary education	17 (56.7)	16 (53.3)		
	None	2 (6.7)	1 (3.3)		

Occupation	Professional	11 (36.7)	13 (43.3)	0.530	0.767
	Manual	17 (56.7)	16 (53.3)		
	None	2 (6.7)	1 (3.3)		

Previous hospitalization	Yes	4 (13.3)	3 (10.0)	0.162	0.688
	No	26 (86.7)	27 (90.0)		

Duration of diabetes	Less than 3 years	0 (0.0)	1 (3.3)	1.017	0.313
	More than 3 years	30 (100.0)	29 (96.7)		

Regular medication use	Yes	27 (90.0)	28 (93.3)	0.218	0.640
	No	3 (10.0)	2 (6.7)		

Family history	Yes	27 (90.0)	28 (93.3)	0.218	0.640
	No	3 (10.0)	2 (6.7)		

*Note:* Values are *n* (%). Between‐group comparisons were conducted using Pearson chi‐square tests. No comparison was computed for marital status because all participants were married.

### 3.2. Neuropathy Symptoms (NTSS‐6)

At baseline, NTSS‐6 symptom‐severity categories did not differ significantly between groups (Table 3; *χ* ^2^ = 0.114 and *p* = 0.990). At 1 month, the distribution of NTSS‐6 categories differed significantly between groups (Table 3; *χ* ^2^ = 34.236 and *p* < 0.001; Cramer’s V = 0.76), favouring the intervention group. In the control group at 1 month, 6.7% of participants were classified as having mild neuropathy, 26.7% moderate neuropathy, 46.7% severe neuropathy and 20.0% very severe neuropathy. In contrast, in the intervention group, 63.3% were classified as having mild neuropathy and 36.7% as having moderate neuropathy, with no participants remaining in the severe or very severe categories.

**TABLE 3:** NTSS‐6 symptom‐severity categories at baseline and 1 month by group.

Timepoint	NTSS‐6 category	Control *n* (%)	Intervention *n* (%)	*χ* ^2^	*p*
Baseline	Scores 0–6: Mild neuropathy	2 (6.7)	2 (6.7)	0.114	0.990
	Scores 7–12: Moderate neuropathy	8 (26.7)	8 (26.7)		
	Scores 13–18: Severe neuropathy	13 (43.3)	14 (46.7)		
	Scores 19–21: Very severe neuropathy	7 (23.3)	6 (20.0)		

One month	Scores 0–6: Mild neuropathy	2 (6.7)	19 (63.3)	34.236	< 0.001
	Scores 7–12: Moderate neuropathy	8 (26.7)	11 (36.7)		
	Scores 13–18: Severe neuropathy	14 (46.7)	0 (0.0)		
	Scores 19–21: Very severe neuropathy	6 (20.0)	0 (0.0)		

*Note:* Values are *n* (%). NTSS‐6 = Neuropathy Total Symptom Score‐6. Between‐group comparisons were conducted using Pearson chi‐square tests. Cramer’s V at 1 month = 0.76.

### 3.3. Diabetes Foot Assessment/Risk Screening

Baseline Diabetes Foot Assessment/Risk Screening categories were similar in the two groups (Table 4; *χ* ^2^ = 0.074, *p* = 0.964). At 1 month, the distribution differed significantly between groups (Table 4; *χ* ^2^ = 30.762, *p* < 0.001; Cramer’s V = 0.72), again favouring the intervention group. In the control group at 1 month, 6.7% of the participants were classified as lower risk, 36.7% as higher risk and 56.7% as having a self‐care knowledge deficit. In the intervention group, 63.3% were classified as lower risk, 36.7% as higher risk and no participants were classified as having a self‐care knowledge deficit.

**TABLE 4:** Diabetes Foot Assessment/Risk Screening categories at baseline and 1 month by group.

Timepoint	Risk category	Control *n* (%)	Intervention *n* (%)	*χ* ^2^	*p*
Baseline	Lower risk	2 (6.7)	2 (6.7)	0.074	0.964
	Higher risk	11 (36.7)	12 (40.0)		
	Self‐care knowledge deficit	17 (56.7)	16 (53.3)		

One month	Lower risk	2 (6.7)	19 (63.3)	30.762	< 0.001
	Higher risk	11 (36.7)	11 (36.7)		
	Self‐care knowledge deficit	17 (56.7)	0 (0.0)		

*Note:* Values are *n* (%). Between‐group comparisons were conducted using Pearson chi‐square tests. Cramer’s V at 1 month = 0.72.

## 4. Discussion

### 4.1. Principal Findings

This quasiexperimental study found that adding a nurse‐led foot‐ankle flexibility and resistance exercise programme to usual care was associated with improved neuropathy symptom‐severity categories and more favourable Diabetes Foot Assessment/Risk Screening categories at 1 month. The between‐group differences were large (Cramer’s V = 0.76 for NTSS‐6 and V = 0.72 for foot risk/self‐care screening), but these estimates were derived from unadjusted categorical analyses in a nonrandomized design and, therefore, should be interpreted as associations rather than causal effects.

### 4.2. Comparison With Prior Evidence

The findings are broadly consistent with growing evidence that exercise interventions can improve symptoms and function in people with DPN [24–26]. A randomized controlled trial of a web‐based foot‐ankle exercise programme demonstrated improvements in ulcer risk factors in people with diabetic neuropathy [13], and intrinsic foot muscle strengthening has been associated with improved ankle mobility and symptom outcomes [12]. In addition, small clinical reports have described benefits of structured foot exercise education for improving circulation and preventive behaviours [27, 28]. The present study extends this literature by providing pragmatic nursing‐context evidence from routine outpatient care, while remaining less definitive than a randomized trial.

### 4.3. Mechanisms

Several plausible mechanisms may explain these improvements. Targeted mobility and strengthening exercises may enhance ankle and foot range of motion, improve muscle activation and support more favourable plantar loading. Regular physical activity is also associated with less severe neuropathy in observational data, reinforcing the importance of movement‐based strategies [29]. In parallel, engaging participants in structured education and supervised practice may strengthen self‐efficacy and adherence to daily foot self‐care behaviours, which are central elements of diabetic‐foot prevention programmes [3, 8, 30]. These mechanisms were not measured directly in the present study, so they should be viewed as explanatory hypotheses rather than confirmed pathways.

### 4.4. Interpretation in the Context of Ulcer Prevention

This study evaluated neuropathy symptoms and risk‐screening indicators rather than ulcer incidence. Recent systematic reviews indicate that foot‐ankle exercise programmes can improve joint mobility and neuropathy signs/symptoms but may not reduce the risk of a first foot ulcer in the short term [14, 15, 31]. Accordingly, our findings should be interpreted as evidence of improvement in intermediate, modifiable clinical indicators rather than as direct proof of ulcer prevention.

### 4.5. Implications for Nursing Practice

These findings have practical implications for nursing care in diabetes clinics. Nurses can integrate brief foot‐risk screening with structured education and a simple exercise demonstration package as part of routine visits. A feasible approach includes (a) baseline foot‐risk screening; (b) short, skill‐based sessions teaching foot‐ankle range‐of‐motion and strengthening exercises; (c) provision of culturally appropriate written/visual materials and (d) low‐cost follow‐up (e.g., telephone calls) to reinforce adherence. Because the intervention was pragmatic and low‐cost, it may be suitable as an adjunct to usual care; however, it should complement rather than replace guideline‐based foot surveillance, footwear assessment and referral pathways for patients at higher risk.

### 4.6. Strengths and Limitations

Strengths include a pragmatic, nurse‐led intervention delivered within routine outpatient care and the use of a validated neuropathy symptom measure. Key limitations are the nonrandom allocation (risk of selection bias), the modest sample size, reliance on unadjusted categorical analyses, use of a study‐specific foot‐risk/self‐care measure without full psychometric evaluation, lack of objective adherence measures and 1‐month follow‐up without measurement of incident ulcers. Future studies should include randomization where feasible, longer follow‐up, adjusted analyses and objective adherence/function measures.

### 4.7. Future Research

Future studies should use randomized designs where feasible, include longer follow‐up and incorporate adjusted longitudinal analyses that account for repeated measurements and potential confounding. Objective measures of adherence and foot function (e.g., plantar pressure, gait metrics, wearable activity data or observed exercise fidelity) would strengthen inference. Research that evaluates ulcer incidence, cost‐effectiveness and implementation outcomes (acceptability, fidelity and sustainability) would further inform nursing practice and policy.

## 5. Conclusion

A nurse‐led programme of foot‐ankle flexibility and resistance exercises was associated with improved neuropathy symptom categories and diabetic‐foot risk/self‐care screening status over 1 month among adults with diabetes. These findings support the promise and feasibility of integrating structured foot‐ankle exercise education into routine outpatient diabetes care, but conclusions should remain cautious because the study was nonrandomized, used exploratory unadjusted categorical analyses and did not measure incident ulcers.

## Funding

No funding was received for this manuscript.

## Conflicts of Interest

The authors declare no conflicts of interest.

## Data Availability

The data that support the findings of this study are available on request from the corresponding author.

## Appendix 1 Diabetic‐Foot Risk Screening/Self‐Care Items

• History of previous foot ulcers or amputation (yes/no)
• Loss of protective sensation or numbness/tingling (yes/no)
• Foot deformity (yes/no)
• Callus or skin breakdown/fissures (yes/no)
• Foot infection signs (yes/no)
• Poor pedal pulses/perfusion concerns (yes/no)
• Inappropriate footwear (yes/no)
• Does not inspect feet daily (yes/no)
• Does not dry between toes/moisturize appropriately (yes/no)
• Does not seek care promptly for foot problems (yes/no)
